# Supplementary material for: Economic expenditures by recreational anglers in a recovering atlantic bluefin tuna fishery
Source: PLoS One. 2022 Aug 4;17(8):e0271823. doi: 10.1371/journal.pone.0271823 (PMC9352073; doi:10.1371/journal.pone.0271823)
Supplement: S1 File — (DOCX) [file pone.0271823.s001.docx]

Supplementary materials

(translated from Danish)

This survey is addressed to everyone who participated as volunteer anglers in the 2020 bluefin tuna tagging project led by DTU Aqua. The survey must be filled out and submitted via email to krmaa@aqua.dtu.dk as soon as possible after the fishing activities are complete.

| **Contact information** |  |
| --- | --- |
| Name |  |
| Phone # |  |
| E-mail |  |
| Zip code |  |
| Age |  |
| Annual income before taxes (not mandatory) |  |
| Gender |  |
| Which boat did you primarily fish from? |  |
| Are you the owner or co-owner of the boat? |  |
| If you are the owner or co-owner of the boat, what is the total value of the boat including boat, trailer, tuna fishing gear, electronics and equipment? |  |
| What is your ownership share in percent (%)? |  |
| How many kilometers have you driven in total due to participation in tuna fishing? |  |
| Which model of car did you use for this purpose? |  |
| **Angling** |  |
| Have you specifically targeted other fish e.g. cod, sea trout, salmon, saithe or flounders during your participation in the tuna tagging project in 2020? |  |
| If so, how many days have you specifically targeted other species than bluefin tuna? |  |
| How many days have you specifically targeted bluefin tuna in 2020? |  |
| How many days have you been prevented from fishing bluefin tuna due to bad weather, lack of tags etc. in 2020. |  |
| How many anglers were fishing with you on average per trip in 2020? |  |
| How many nights have you spent in the area around Skagen as a direct consequence of fishing for bluefin tuna in 2020? |  |
| Was there any other purpose with your stay in or near Skagen in 2020? E.g. family visits, sightseeing etc. |  |
| If so, how large a proportion in percent of your stay in or near Skagen was related to these other purposes? |  |
| How many hours have you spent on preparation and transport directly related to bluefin tuna fishing before arriving in Skagen in 2020? H |  |
| How many hours have you on average spent per fishing day including sailing time directly related to bluefin tuna fishing in 2020? |  |
| How many bluefin tuna did you catch and transfer to the tagging boat or release without tagging in 2020? |  |
| What was the length (CFL) in cm and weight of the tunas you caught in 2020? |  |
| How many of the tunas you transferred to the tagging boat or released yourself died during the fight, tagging or release in 2020? |  |
| How many nautical miles have you sailed because of tuna fishing in 2020? |  |
| Would you say that you have eaten out on restaurants or similar significantly more during the tuna project than you would have if you did not participate in the tuna fishing in 2020? |  |
| **Expenditures related to tuna fishing**  How much money have you personally spent on: |  |
| Fishing gear including rods and reels directly related to tuna fishing in 2020? |  |
| Other fishing equipment including line, hooks, leader materials, fight belts etc. directly related to tuna fishing in 2020? |  |
| Bait, chum and ice directly related to tuna fishing in 2020? |  |
| Boat expenses, insurance, engine and service directly related to tuna fishing in 2020? |  |
| Boat equipment including electronics, rod holders, chum-machine, fight-chair etc. directly related to tuna fishing in 2020? |  |
| Mooring, harbor expenses, facilities in the harbor and boat ramp directly related to tuna fishing in 2020? |  |
| Other equipment including clothing, gloves, glasses, camera, binoculars etc. directly related to tuna fishing in 2020? |  |
| Fuel directly related to tuna fishing in 2020? |  |
| Accommodation directly related to tuna fishing in 2020? |  |
| Other transport expenses including bridge and highway tolls, ferry, boat transport, parking, car rental etc. directly related to tuna fishing in 2020? |  |
| Public transportation directly related to tuna fishing in 2020? |  |
| Souvenirs, merchandise including team shirts, keyrings, trophies, flags etc. directly related to tuna fishing in 2020? |  |
| Food and drinks including restaurants and bars in Skagen directly related to tuna fishing in 2020? |  |
| Other expenses directly related to tuna fishing in 2020? |  |

Thank you very much for your participation!

**Contact**

Kristian Maar

DTU Aqua - Technical University of Denmark

E-mail: [krmaa@aqua.dtu.dk](mailto:krmaa@aqua.dtu.dk)
